# Deficiency of MMP-10 Aggravates the Diseased Phenotype of Aged Dystrophic Mice

**DOI:** 10.3390/life11121398

**Published:** 2021-12-14

**Authors:** Arantxa Baraibar-Churio, Míriam Bobadilla, Florencio J. D. Machado, Neira Sáinz, Carmen Roncal, Gloria Abizanda, Felipe Prósper, Josune Orbe, Ana Pérez-Ruiz

**Affiliations:** 1 Regenerative Medicine Program, Foundation for Applied Medical Research (FIMA), University of Navarra, 31009 Pamplona, Spain; abaraibarc@alumni.unav.es (A.B.-C.); mbobadillam@alumni.unav.es (M.B.); nsainz@unav.es (N.S.); gabizanda@unav.es (G.A.); fprosper@unav.es (F.P.); 2 Instituto de Investigación Sanitaria de Navarra (IdiSNA), 31008 Pamplona, Spain; fflorenciom@alumni.unav.es (F.J.D.M.); croncalm@unav.es (C.R.); josuneor@unav.es (J.O.); 3 Laboratory of Atherothrombosis, Program of Cardiovascular Diseases, University of Navarra, 31009 Pamplona, Spain; 4 Centre for Nutrition Research, University of Navarra, 31008 Pamplona, Spain

**Keywords:** matrix metalloproteinases, muscular dystrophy, skeletal muscle, cardiac muscle

## Abstract

Matrix metalloproteinases (MMPs) have been implicated in the progression of muscular dystrophy, and recent studies have reported the role of MMP-10 in skeletal muscle pathology of young dystrophic mice. Nevertheless, its involvement in dystrophin-deficient hearts remains unexplored. Here, we aimed to investigate the involvement of MMP-10 in the progression of severe muscular dystrophy and to characterize MMP-10 loss in skeletal and cardiac muscles of aged dystrophic mice. We examined the histopathological effect of MMP-10 ablation in aged *mdx* mice, both in the hind limb muscles and heart tissues. We found that MMP-10 loss compromises survival rates of aged *mdx* mice, with skeletal and cardiac muscles developing a chronic inflammatory response. Our findings indicate that MMP-10 is implicated in severe muscular dystrophy progression, thus identifying a new area of research that could lead to future therapies for dystrophic muscles.

## 1. Introduction

Duchenne muscular dystrophy (DMD) is the most devastating X-linked muscular disorder, affecting approximately 1 in 3500 newborn males [1]. Mutations in the dystrophin gene result in a complete loss of the dystrophin protein or shorter or partially functional dystrophin. Dystrophin links the myofiber cytoskeleton to the extracellular matrix (ECM). Consequently, dystrophic muscles are more susceptible to muscular mechanical injury and sarcolemma damage, with myofibers undergoing continuous cycles of injury/repair. Under this chronic scenario, damage overcomes the regenerative potential of the muscle, contributing to progressive muscle wasting [1]. Beyond skeletal muscle deterioration, DMD patients suffer serious heart complications, which are responsible for premature death [2]. However, cardiac involvement in disease progression has not been studied as extensively as skeletal muscle disease, and therapeutic options for DMD cardiac dysfunction are still limited. 

MMPs are a family of 25 proteolytic enzymes that can degrade various components of the connective and fibrous tissues that compose the ECM, participating in its remodeling during several physiological and pathological processes [3]. Increasing evidence suggests the participation of MMPs in muscular dystrophy progression, with gene expression and activation of various MMPs deregulated in dystrophic skeletal muscles being found in both DMD patients and animal models of muscular dystrophy [4]. Nevertheless, there is relatively little knowledge regarding the implications of MMPs in severe muscular dystrophy and considerably less information related to their role in the cardiac failure associated with DMD. MMP-10 belongs to the stromelysin family of MMPs, and it plays an essential role in skeletal muscle maintenance and regeneration [5,6,7]. In mice, healthy skeletal muscles express MMP-10, and protein levels increase in injured and dystrophic muscles [5,6,7]. Interestingly, genetic ablation of MMP-10 significantly impairs the deteriorated muscle phenotype of young *mdx* mice, a mouse model of DMD, with skeletal muscles exhibiting an altered structure with delayed muscle regeneration after damage [5]. 

Due to the critical role that MMP-10 plays during muscular dystrophy progression and the consequences of MMPs deregulation during cardiac remodeling [8], we aimed to better understand the biological function of MMP-10 in dystrophic mice. To this end, we histologically analyzed skeletal and cardiac muscles of aged *mdx* mice to assess the implication of MMP-10 ablation in severe muscular dystrophy. 

## 2. Materials and Methods

### 2.1. Mice

B6Ros.Cg-Dmdmdx-5cv/J (*mdx*) mice were obtained from the Jackson Laboratory, while MMP-10 KO mice were provided by Dr. WC Parks (University of Washington, Seattle, WA, USA). Male MMP-10 KO mice were crossed with female homozygous *mdx* mice to generate the *mdx*;MMP-10 KO animals. All genotypes were determined by PCR analysis of mouse ear DNA using specific primers for dystrophin (forward, 5′-TTCATTGATGGAGACGGAA-3′; reverse, 5′-TGAGCATGAAACTGTTCTTTCA-3′), and MMP-10 (forward primer 1, 5′-TGTGTAGTGCCTACACTAAGCCA-3′; forward primer 2, 5′-TGCCTCGTCCTGCAGTTCATTC-3′; reverse primer, 5′-TAAGGGTGTGAGTCTTCATGGAT-3′). C57BL/6J wild-type animals were purchased from Harlan and were used as controls. All animals were housed in the animal housing facilities at the University of Navarra, according to current legislation. Only male mice were used in this study since muscular dystrophy is an X-linked muscular human disease.

### 2.2. Tissue Immunostaining and Image Capture and Quantification 

Skeletal muscles were frozen in isopentane cooled in liquid nitrogen. Hearts were fixed in 4% paraformaldehyde, washed in PBS and 70% ethanol and finally embedded in paraffin. Serial 9 μm cryosections and 10 μm heart sections were collected at 200 μm intervals through the entire tissues. Sections were blocked with 20% goat serum and immunostained with mouse anti-eMyHC (DSHB, Iowa city, IA, USA; F1.652 clone), anti-CD45 (Biolegend, San Diego, CA, USA; 30F-11 clone; Ref.: 103102), anti-NIMP-R14 (Abcam; Ref.: ab2557), rabbit anti-laminin (Sigma, St. Louis, MO, USA; Ref.: L9393), anti-MMP10 (Abcam, Cambridge, UK; Ref.: ab38930) or Cy3-labeled goat anti-mouse IgG (Invitrogen). When required, fluorochrome-conjugated secondary antibodies (Molecular Probes) were applied. DAPI was used to stain the nuclei. Cardiac sections were stained for rabbit anti-F480 (Serotec, Hercules, CA, USA; Ref.: NCA497GA), followed by visualizing with diaminobenzidine. Sirius Red and H&E stainings were performed as previously described [5].

Immunostained and stained tissue sections were viewed using a Zeiss Axiophot epi-fluorescence or a Zeiss Axio Imager M1 microscope, and digital images were acquired with an AxioCamMR3 or AxioCamICc3 camera (Zeiss, Oberkochen, Germany). Tissue section analyses, including the whole tissue transversal sections, were performed using the AxioVision and ImageJ software. Positive staining was calculated as the percentage of the stained area, dividing the positive area by the whole area of the tissue analyzed. Morphometry determined the number of fibers and the cross-sectional area (µm^2^) of each fiber after laminin immunostaining. 

### 2.3. Quantitative Real-Time PCR

Total RNA was isolated from cardiac muscles and purified using Trizol (Ambion). Total RNA was reverse transcribed using Superscript II RNAse H reverse transcriptase (Invitrogen, Waltham, MA, USA), and the cDNA was amplified using the TaqManH Universal PCR Master Mix (Applied Biosystems, Waltham, MA, USA). Transcript levels were quantified by real-time PCR (QuantStudioTM 5 Real-Time PCR System, Applied Biosystems, Waltham, MA, USA) under the following conditions: 95 °C for 3 min, followed by 40 cycles consisting of 60 s at 90 °C, 60 s at 60 °C and 60 s at 72 °C. Specific primers and probes were designed (Primer3 program; version 0.4.0) for Mmp2 (forward, 5′-ATGGCAAGTATGGCTTCTGC-3′; reverse, 5′-GTAGGAGGTGCCCTGGAAG-3′; probe, 5′-AGCCTTGTTTACCATGGGTG-3′), Mmp3 (forward, 5′-CGATGATGAACGATGGACAG-3′; reverse, 5′-AAGTTCATGAGCAGCAACCA-3′; probe, 5′-AGGATGTCACTGGTACCAACCT-3′), Mmp9 (forward, 5′-AGACGACATAGACGGCATCC-3′; reverse, 5′-GTGGTTCAGTTGTGGTGGTG-3′; probe, 5′-GTCGTGGCTCTAAGCCTGAC-3′), col10a1 (Ref.: Mm00487041_m1; ThermoFisher Scientific, Waltham, MA, USA) and Actb (forward, 5′-GACGGCCAGGTCATCACTAT-3′; reverse, 5′- CTTCTGCATCCTGTCAGCAA-3′). For amplification of fibrotic and cardiac genes, the cDNA was amplified using PowerUp SYBR Green Master Mix (Applied Biosystems, Waltham, MA, USA). Specific primers were used for Cthrc1 (forward, 5′- GCTGTCAGCGCTGGTATTTT-3′; reverse, 5′-ACCCAGATGGCCACATCTAC-3′), Col1a1 (forward, 5′-AGGCGAAGGCAACAGTCG-3′; reverse, 5′- TTTACACGAAGCAGGCAGGG-3′), Lox (forward, 5′- CCCGACCCCTACTACATCCA-3′; reverse, 5′- AGTCTCTGACATCCGCCCT-3′), Postn (forward, 5′-TTCGTGGCAGCACCTTCAAA-3′; reverse, 5′- GTCACCGTTTCGCCTTCTTT-3′), Myh6 (forward, 5′- ATGTTAAGGCCAAGGTCGTG-3′; reverse, 5′-CACCTGGTCCTCCTTTATGG-3′), Tpn (forward, 5′-CTGAGACAGAGGAGGCCAAC-3′; reverse, 5′- TTCTCGAAGTGAGCCTCGAT-3′) and Gapdh (forward, 5′- CTCCCACTCTTCCACCTTCG-3′; reverse, 5′-GCCTCTCTTGCTCAGTGTCC-3′). All measurements were normalized to Actb or Gapdh. 

### 2.4. Statistical Analysis

All statistical analyses were performed using SPSS 15.0 (SPSS Inc.). The Kolmogorov–Smirnov test was used to test whether measures were normally distributed. Variables were analyzed with the Mann–Whitney U test or Student *t* tests. All experiments were performed using at least three mice per condition. Data are expressed as means ± SEM. *p* values < 0.05 were considered to be statistically significant.

## 3. Results

### 3.1. MMP-10 Deficiency Decreases Lifespan of Dystrophic Mice 

**Figure 1:**
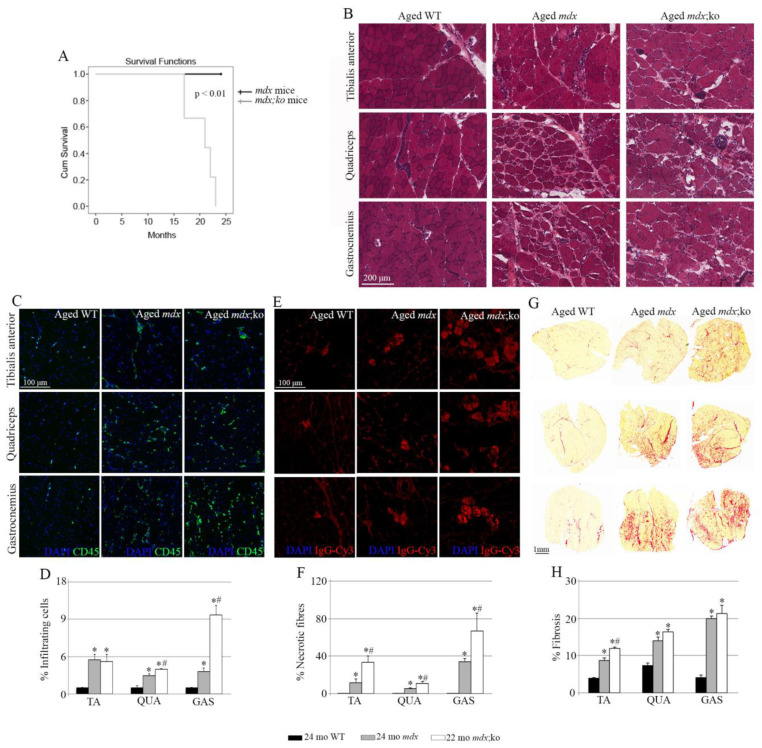
Loss of MMP-10 precipitates *mdx* mice death and deteriorates the skeletal muscles of aged *mdx* mice. (**A**) Graph shows the survival rates between mouse genotypes. Representative hematoxylin–eosin (**B**), CD45 (**C**); quantified in (**D**), Cy3-IgG (**E**); quantified in (**F**) and Sirius red (**G**); quantified in (**H**) stainings of tibialis anterior, quadriceps and gastrocnemius muscles isolated from aged wild-type *mdx* and *mdx*;MMP-10 KO (*mdx*;ko) mice showing infiltrating cells, necrotic fibers and fibrosis, respectively. DAPI was used to visualize the nuclei. All data in *mdx* and *mdx*;ko mice were related to those from WT mice and are expressed as fold change. Values are presented as mean ± SEM from three aged wild-type mice at 24 months of age, four 24-month-old *mdx* mice and four *mdx*;ko mice at 22 months of age, where * and ^#^ denote statistically significant differences between wild-type and dystrophic mice, and *mdx* and *mdx*;MMP-10 KO animals, respectively (*p* < 0.05). Abbreviations: KO, knockout; *mdx*;ko, *mdx*;MMP-10 KO; WT, wild type; mo, months; TA, tibialis anterior; QUA, quadriceps; GAS, gastrocnemius.

In order to evaluate the implications of MMP-10 in serious muscular dystrophy, *mdx* mice lacking MMP-10 were left to age. Compared with aged *mdx* animals, deficiency of MMP-10 compromised the animal´s welfare, with some animals dying at the age of 17 months. After noticing first signs of physical discomfort, such as slack posture, lack of mobility, abnormal field behavior and marked kyphosis, most aged MMP-10-deficient *mdx* mice were humanely sacrificed to avoid pain and suffering (Figure 1A). On the contrary, *mdx* mice lived longer and were sacrificed at the age of 24 months. Thus, deficiency of MMP-10 significantly decreased survival rates of dystrophic mice.

### 3.2. Ablation of MMP-10 Deteriorates The Skeletal Muscle Dystrophic Phenotype of Aged mdx Mice 

We previously showed that MMP-10 protein levels increase in adult dystrophic muscles, compared with adult wild-type mice [5]. Now, we further addressed the participation of MMP-10 in muscular dystrophy progression by extending our previous data to aged animals. We found by immunostaining that the MMP-10 protein content was significantly higher in skeletal muscles from 24-month-old *mdx* mice than in muscles from 2-month-old dystrophic mice (Figure S1A,B). 

**Figure 2:**
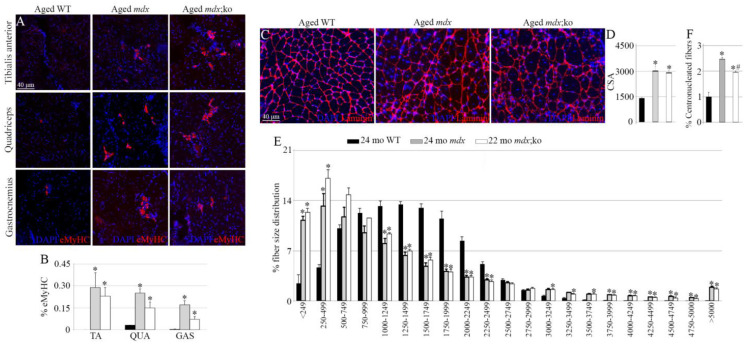
MMP-10 ablation does not impair the ongoing muscle regeneration of aged *mdx* mice. Representative images of muscles immunostained for eMyHC (**A**); quantified in (**B**) and laminin (**C**) in aged wild-type, *mdx* and *mdx*;MMP-10 KO mice. Average cross sectional area (CSA) of the fibers (**D**), fiber size distribution (**E**) and centrally nucleated fibers (**F**) in aged mouse strains. DAPI was used to visualize all the nuclei. In graphs B and F, measurements in aged *mdx* and aged *mdx*;ko mice were related to those from aged WT mice and are expressed as fold change. Values are presented as mean ± SEM from three 24-month-old wild-type mice, four 24-month-old *mdx* mice and four *mdx*;ko mice at 22 months of age. * denotes a statistically significant difference from wild-type and dystrophic mice, while ^#^ defines significance between *mdx* and *mdx*;MMP-10 KO animals (*p* < 0.05). Abbreviations: KO, knockout; WT, wild type; *mdx*;ko, *mdx*;MMP-10 KO; mo, months; TA, tibialis anterior; QUA, quadriceps; GAS, gastrocnemius.

To assess the role of MMP-10 in severe muscular dystrophy, we analyzed the consequences of MMP-10 ablation in aged *mdx* mice. To this end, the tibialis anterior (TA), quadriceps and gastrocnemius muscles were isolated from 24-month-old *mdx* mice and *mdx*;MMP-10 KO mice at 22 months of age, including a control group of 24-month-old wild-type mice. We first probed the absence of dystrophin protein from muscles of aged dystrophic strains by immunostaining (Figure S1C). Comparisons between muscles from aged wild-type, *mdx* and MMP-10-deficient *mdx* mice showed microstructural differences, with muscles from aged dystrophic strains exhibiting a pronounced deteriorated muscle phenotype (Figure 1B). Aged MMP-10-deficient dystrophic muscles had areas of massive muscle degeneration and moderate cellular infiltration, with a higher presence of necrotic fibers and fatty infiltration, in comparison with the skeletal muscles from aged *mdx* mice (Figure 1B). Immunostaining for CD45 confirmed the presence of infiltrating cells in muscles from aged dystrophic mice, with a significant increase in the quadriceps and gastrocnemius of aged MMP-10-deficient *mdx* mice, compared with *mdx* animals (Figure 1C,D). By using a mouse IgG-Cy3 antibody, we verified that the dystrophic condition significantly increased muscle necrosis, as monitored by intracellular fiber staining for IgG (Figure 1E,F), with loss of MMP-10 further deteriorating the dystrophic phenotype. The TA muscles from aged MMP-10-deficient *mdx* mice accumulated twice as many necrotic myofibers compared with aged *mdx* animals, while the quadriceps and gastrocnemius had slightly significantly larger necrotic areas (Figure 1E,F). Aged dystrophic muscles accumulated more connective tissue than muscles from 24-month-old wild-type mice (Figure 1G,H). Surprisingly, comparisons between aged *mdx* and aged *mdx*;MMP-10 KO mice showed that ablation of MMP-10 increased fibrotic accumulation only in the TA muscles (Figure 1G,H). As expected, a rare eMyHC^+^ immature myofiber was detected in muscles from aged wild-type mice, in comparison with muscles from aged dystrophic strains (Figure 2A,B). However, deficiency of MMP-10 did not impair ongoing regeneration in dystrophic muscles of aged mice (Figure 2A,B). 

Morphometric analysis after immunostaining quadriceps and gastrocnemius tissue sections for laminin showed that MMP-10 ablation did not modify the average cross sectional area of the myofibers of aged *mdx* mice or the fiber size distribution of muscles (Figure 2C–E). However, the hypertrophic muscle response associated with dystrophic disease was evident when comparing aged dystrophic and aged wild-type mice (Figure 2C–E). We next assessed the number of centrally nucleated myofibers as an index of muscle degeneration and regeneration. When compared with aged wild-type mice, centro-nucleated myofibers significantly increased in aged dystrophic strains but MMP-10 loss reduced this effect (Figure 2F), suggesting that *mdx*;MMP-10 KO mice may have a faster degeneration–regeneration cycle than *mdx* mice. 

Overall, our data suggest that MMP-10 deficiency aggravates the dystrophic phenotype of aged *mdx* mice by generating a chronic inflammatory response with increased fiber necrosis, without further altering muscle structure.

### 3.3. MMP-10 Ablation Leads to Chronic Inflammation in Dystrophin-Deficient Hearts

To address the participation of MMP-10 in severe muscular dystrophy, we examined by immunostaining the MMP-10 protein levels in hearts from young and aged wild-type and *mdx* mice, finding that its abundance significantly increased during physiological aging and disease progression (Figure S2). 

**Figure 3:**
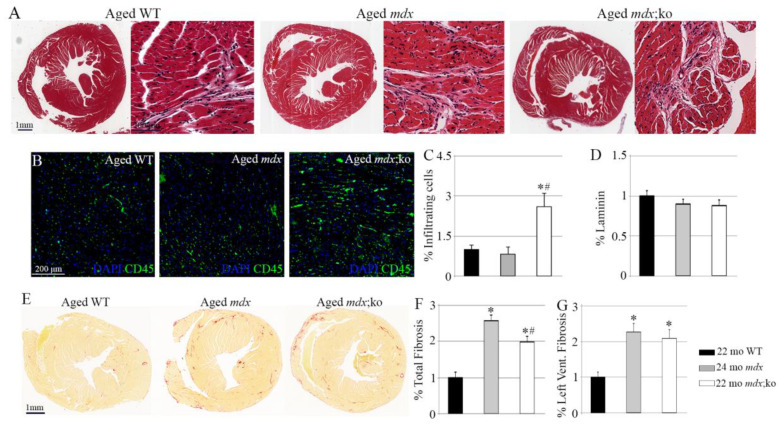
MMP-10 deficiency affects cardiac muscle composition in aged *mdx* mice. Representative hematoxylin–eosin staining of transversal heart sections isolated from aged wild-type, *mdx* and *mdx*;MMP-10 KO mice (**A**). Representative images of CD45 (**B**); quantified in (**C**) immunostaining showing infiltrating cells in hearts from wild-type, *mdx* and *mdx*;MMP-10 KO mice. The graph shows quantification of laminin expression in cardiac tissue sections (**D**). Representative images of cardiac muscles stained with Sirius red (**E**) and graphs showing fibrosis accumulation in total cardiac tissue sections (**F**) and in the left ventricular area (**G**) in aged animals. DAPI was used to visualize nuclei. All data in *mdx* and *mdx*;ko mice were related to those from WT mice and are expressed as fold change. Values are presented as mean ± SEM from three 22-month-old wild-type, four 24-month-old *mdx* and four 22-month-old *mdx*;MMO-10 KO independent *mice*. * denotes a statistically significant difference from wild-type and dystrophic mice; ^#^ identifies statistically differences between aged *mdx* and *mdx*;MMP-10 KO animals (*p* < 0.05). Abbreviations: KO, knockout; WT, wild type; mo, months; *mdx*;ko, *mdx*;MMP-10 KO.

To investigate the role of MMP-10 in severe muscular disease, we analyzed the consequences of MMP-10 ablation in hearts from aged *mdx* mice. Absence of dystrophin from cardiac tissues of *mdx* strains was first confirmed by immunostaining (Figure S3A). We next assessed changes in the hearts of aged wild-type and *mdx* mouse strains at the microstructural level, finding that ablation of MMP-10 significantly increased the presence of infiltrating cells in dystrophic hearts (Figure 3A–C). To address the origin of cell infiltration, cardiac muscles were immunostained for NIMPR14 and F4/80 in order to detect the presence of neutrophils and macrophages, respectively. Few neutrophils were detected in aged hearts, with no statistical differences being found between wild-type, *mdx* and *mdx*;MMP-10 KO mice (Figure S3B,C). Macrophages were equally localized in the hearts of aged wild-type and *mdx* mice. However, ablation of MMP-10 doubled the presence of macrophages in dystrophic hearts (Figure S3D,E). The different presence of neutrophils and macrophages may state differences between acute and chronic inflammatory processes, highlighting disease chronicity in aged *mdx*;MMP-10 animals. 

Cardiac fibers were not modified by deficiency of dystrophin and MMP-10, with hearts from aged mice showing similar laminin structure and expression levels (Figure 3D). Furthermore, mRNA levels of cardiac myosin and troponin were equally expressed in hearts from aged *mdx*;MMP-10 KO, *mdx* and wild-type mice (Figure S4A), suggesting that cardiac function is not affected by dystrophin and MMP-10 ablation. As expected, the dystrophic condition increased the abundance of fibrotic tissue in cardiac muscles (Figure 3E–G), compared with muscles of aged wild-type origin. However, hearts from aged MMP-10-deficient dystrophic mice accumulated less fibrotic tissue than hearts from aged *mdx* mice (Figure 3F,G), but these differences were not related to the fibrosis developed in the left ventricular areas (Figure 3E–G), which usually are more affected in DMD patients. To further address the decrease in fibrotic tissue accumulation in *mdx*;MMP-10 KO hearts in comparison with *mdx* and wild-type tissues, we analyzed the expression of genes associated with cardiac fibrosis, including conventional genes, such as *Col1a1*, as well as genes implicated in earlier and later processing of collagen, such as *Cth2c1*, *Postn* and *Lox* [9,10]. No differences in *Col1a1* expression levels were found between aged mouse strains (Figure S4B). Compared with aged wild-type mice, expression of *Cthc1* and *Lox* was increased in hearts from aged dystrophic mice, and this effect was attenuated by MMP-10 ablation (Figure S4B). Furthermore, loss of MMP-10 from cardiac dystrophic muscles reduced *Postn* gene expression (Figure S4B). We further analyzed the expression levels of *Col10a1* in order to assess fibrosis calcification and ossification in cardiac muscles since MMP-10 may participate in heterotopic bone formation [11], finding no differences between aged *mdx* and *mdx*;MMP-10 KO mice (Figure S4C). 

Ablation of a specific MMP can lead to compensatory effects, thus we next examined expression of *Mmp3*, which belongs to the stromelysin family, as well as examining MMP-10, *Mmp2* and *Mmp9*, the two major MMP gelatinases that have been associated with DMD cardiomyopathy [12]. We found that MMP-10 loss altered *Mmp3* and *Mmp2* expression in hearts from aged *mdx* mice, avoiding the increase associated with the severe dystrophic condition (Figure S4D). No differences in *Mmp9* mRNA expression levels were found between hearts from aged wild-type, *mdx* and *mdx*;MMP-10 KO mice (Figure S4D).

Taken together, these findings suggest that hearts in aged *mdx*;MMP-10 KO mice exhibited a higher chronic inflammatory response than aged *mdx* mice but without cardiac structure being further affected. 

## 4. Discussion

We analyzed the skeletal and cardiac muscles of aged *mdx* mice, which reproduce more accurately human disease, to assess the involvement of MMP-10 in severe muscular dystrophy. MMP-10 deficiency, apart from compromising the skeletal muscle phenotype of aged *mdx* mice, deteriorates dystrophic hearts, with muscles developing a chronic inflammatory response that may precipitate death. 

Although a tightly regulated, transient inflammatory response is required for normal muscle regeneration, prolonged inflammation promotes dystrophic disease [13]. Increasing evidence shows a complex collaborative cellular activity in muscular dystrophy progression, involving several different cell types [14]. Of these, inflammatory cells have been prominent in triggering fiber necrosis, and their effects on the overall muscle disease in muscular dystrophy are still under investigation [13]. Inflammatory cells secrete cytokines and interleukins that facilitate the myogenic functions of adult muscle stem cells in a complex balance with the activity of neutrophils, eosinophils, lymphoid cells, mast cells, macrophages and muscle-resident fibro-adipogenic progenitors, influencing skeletal muscle responses during muscular disease [14,15,16]. The excessive activity of inflammatory cells leads to fiber necrosis, fibrosis and fatty infiltration in dystrophic muscles. In this sense, it is reasonable to think that changes in the ECM components of the muscles associated with chronic inflammation impact cell mobility and the distribution of cytokines. Therefore, ECM modulators such as MMPs may contribute to cellular instability during disease progression. In line with this idea, we previously reported that genetic ablation of MMP-10 in young *mdx* mice modified the composition of the muscle ECM and that this was accompanied by increased cell infiltration and loss of muscle stem cells, worsening the dystrophic phenotype [5]. Now, we support our previous findings in a context of severe muscular disease.

Although all DMD teenagers have signs of cardiac dysfunction, and up to 40% of patients may die from heart complications [2], cardiac involvement has not been studied as extensively as skeletal muscle disease, and the pathogenesis of dystrophin-deficient cardiomyopathy is not completely understood. Nevertheless, the inflammatory response disrupts heart homeostasis, and the degree of inflammatory cell infiltration in cardiac tissue is strongly associated with disease severity in muscular dystrophies [13], supporting our finding that MMP-10 loss led to severe dystrophic phenotype. MMPs regulate the inflammatory components of the wound healing response to the diseased heart, and through their direct activity on ECM components of the heart or acting as upstream signaling initiators, they may initiate cellular signaling cascades [17]. Thus, ablation of MMP-10 may disrupt cellular signaling in dystrophic hearts, causing serious chronic inflammation.

Our study supports ablation of MMP-10 as being responsible for the premature death of aged dystrophic mice. However, some considerations regarding the consequences of MMP-10 ablation in aged *mdx* mice remain enigmatic. Differences in collagen deposits between skeletal and cardiac fibers in mutant animals and the fact that laminin structure was not affected by MMP-10 ablation were unexpected, especially considering that these ECM components are substrates degraded by MMP-10. Recent evidence demonstrates that ablation of one MMP can be compensated for by other MMP members [18]. Accordingly, activity of MMP-2 increases in MMP-10-ablated muscles [5], and MMP-2 can cleave these ECM components [3]. What is clear is that the in vivo function of the different members of the MMP family is extremely complex and that they may cause opposing and unexpected effects on the progression of different or same diseases [4,8,17], such as those affecting skeletal and cardiac muscle. Furthermore, the complexity in MMP activity increases by cell-specific induction, with some MMPs playing dual roles depending on tissue type and stage of the disease. Here, we found that dystrophic condition and MMP-10 ablation changed the expression of *Mmp3* and *Mmp2* in the hearts of aged mice, while we previously showed that ablation of MMP-10 from young wild-type and *mdx* mice differently affects expression and activity of *Mmp2*, *Mmp3* and *Mmp9* in skeletal muscles. In addition, it remains perplexing that ablation of MMP-10 deteriorates the dystrophic phenotype of aged mice since MMP-10 protein levels increase in skeletal and cardiac muscles of aged *mdx* mice.

Future work aimed at specifying the influence of MMP-10 on the inflammatory processes and its relation to the deterioration of skeletal and cardiac muscles and its connection to other MMP members is required to understand the premature death of ablated dystrophic mice. This includes further investigation into cardiac function and its relation to the diaphragm since loss of respiratory muscle contractility is sufficient to induce heart function failure [19]. This may open up new therapeutic opportunities for DMD patients. 

## Data Availability

Data can be obtained from the corresponding authors on request.

## Supplementary Materials

The following are available online at https://www.mdpi.com/article/10.3390/life11121398/s1, Figure S1: Representative images of gastrocnemius from 2-month-old young (*n* = 4) and 24-month-old mdx (*n* = 4) mice immunostained for MMP-10; Figure S2: Representative hearts of young (1-month-old) and aged (22-month-old) wild type mice and young (1-month-old) and aged (24-month-old) mdx animals immunostained for MMP-10; Figure S3: Representative images of cardiac muscles of 22-month-old wild type mice (*n* = 3), 24-month-old mdx (*n* = 4) and 22-month-old mdx;MMP-10 KO (*n* = 4) mice immunostained for dystro-phin (**A**), NIMP-R14 (**B**）; quantified in （**C**) and F4/80 (**D**); quantified in (**E**); Figure S4: Graphs show gene expression levels of Myh6, Tpn (**A**), Col1a1, Cth2c1, Postn, Lox (**B**), Cola10a1 (**C**) and Mmp3, Mmp2 and Mmp9 (**D**) related to Actb or Gapdh.
