# Supplementary material for: Deficiency of MMP-10 Aggravates the Diseased Phenotype of Aged Dystrophic Mice
Source: Life (Basel). 2021 Dec 14;11(12):1398. doi: 10.3390/life11121398 (PMC8705381; doi:10.3390/life11121398)
Supplement: Supplementary file 1 [file life-11-01398-s001.zip › life-1463545-supplementary.pdf]

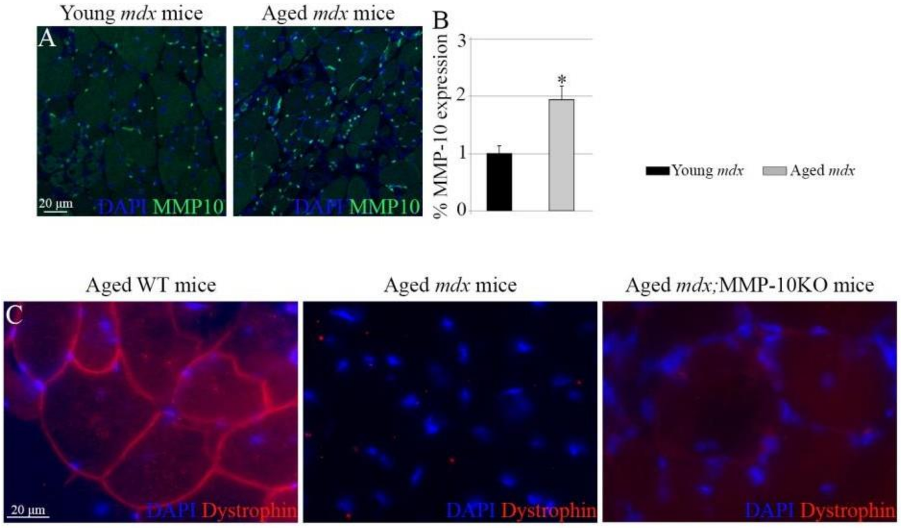

Figure S1. Representative images of gastrocnemius from 2-month-old young (n = 4) and 24-month-old mdx (n = 4) mice immunostained for MMP-10 (**A**); quantified in (**B**). Skeletal muscles from 24- months-old wild type mice, 24-months-old mdx and 22-months-old mdx;MMP-10 KO mice im-munostained for dystro-phin (**C**). Data in mdx;ko mice were related to those from mdx mice, and are expressed as fold change. Data represent the mean ± SEM where # defines significant differences between experimental groups. Abbreviations: KO, knock out; WT, wild type.

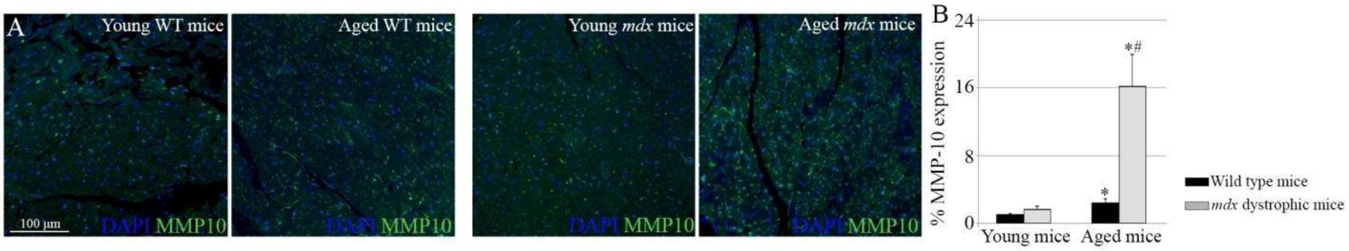

Figure S2. Representative hearts of young (1-month-old) and aged (22-month-old) wild type mice and young (1-month-old) and aged (24-month-old) mdx animals immunostained for MMP-10 (**A**). Graph shows the percentage of positive stained area, where values are expressed as the mean ± SEM of four biologi-cal replicates (**B**). All data were related to those from young WT mice, and are expressed as fold change. Ab-breviations: WT, wild type.

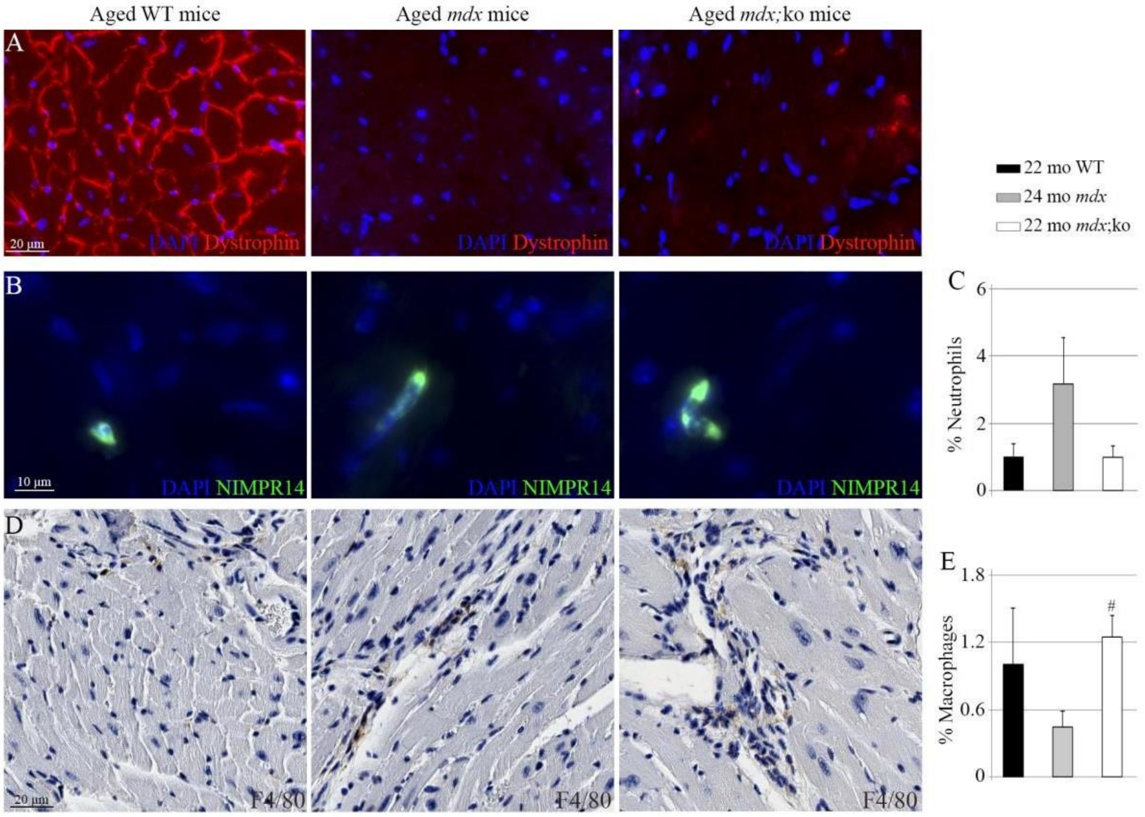

Figure S3. Representative images of cardiac muscles of 22-month-old wild type mice (*n* = 3), 24-month-old mdx (*n* = 4) and 22-month-old mdx;MMP-10 KO (*n* = 4) mice immunostained for dystro-phin (**A**), NIMP-R14 (**B**); quantified in (**C**) and F4/80 (**D**); quantified in (**E**). All measurements in mdx and mdx;ko mice were related to those from WT mice, and are expressed as fold change. Data show the mean ± SEM with # defining significant differences between aged mdx and mdx;MMP-10 KO animals ($p < 0.05$). Abbre-viations: KO, knock out; WT, wild type; mdx;ko, mdx;MMP-10 KO; mo, months.

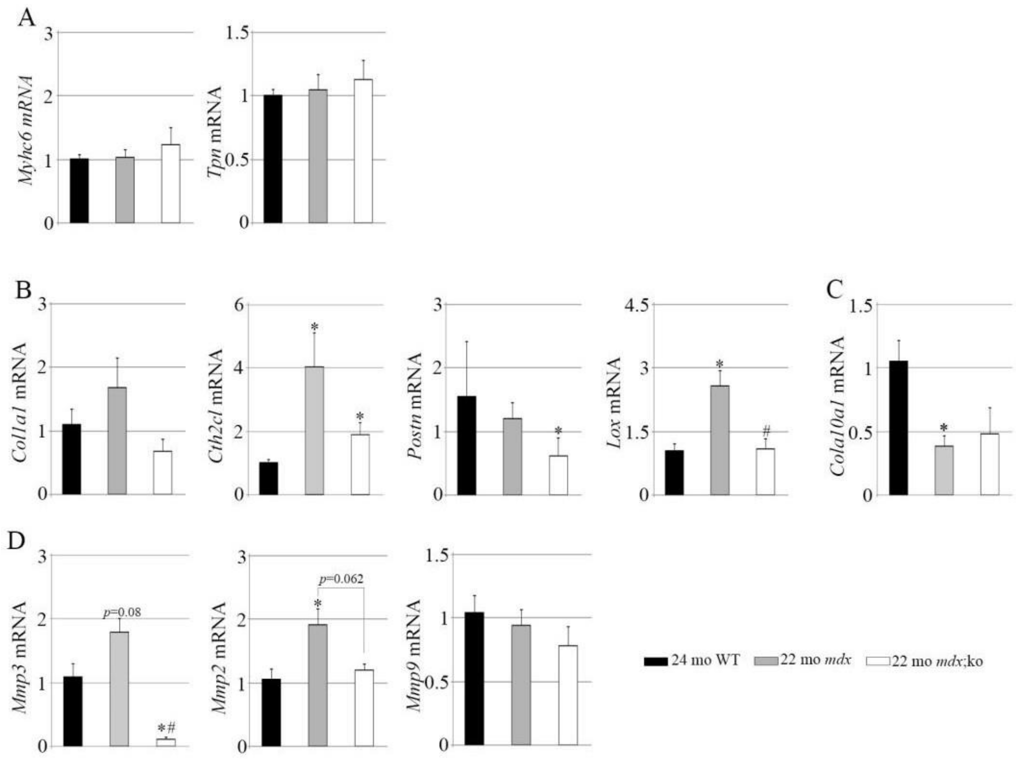

Figure S4. Graphs show gene expression levels of Myh6, Tpn (**A**), Col1a1, Cth2c1, Postn, Lox (**B**), Cola10a1 (**C**) and Mmp3, Mmp2 and Mmp9 (**D**) related to Actb or Gapdh. Measurements in mdx and mdx;MMP- 10 KO mice at 22 months of age were expressed as a fold change compared to 24-month-old wild type controls and expressed as the mean ± SEM of four biological replicates. Significant differences with * and # defining significant differences between experimental groups ($p < 0.05$). Abbreviations: KO, knock out; WT, wild type; mdx;ko, mdx;MMP-10 KO; mo, months.
